# Preparation and Insight of [010]‐Orientated BiVO_4_ Planar Photoanode via One‐Step Pyrolysis for Significantly Promoted Charge Separation and Water Oxidation

**DOI:** 10.1002/advs.202416474

**Published:** 2025-04-02

**Authors:** Sainan Zhang, Jiaming Zhang, Nengcong Yang, Yejun Xiao, Rong Wang, Yunfeng Bao, Guijun Ma, Shengye Jin, Fuxiang Zhang

**Affiliations:** ^1^ Department of Chemical Physics University of Science and Technology of China Hefei Anhui 230026 P. R. China; ^2^ State Key Laboratory of Catalysis Dalian Institute of Chemical Physics Dalian National Laboratory for Clean Energy Chinese Academy of Sciences Dalian Liaoning 116023 P. R. China; ^3^ School of Physical Science and Technology ShanghaiTech University Shanghai 201210 P. R. China; ^4^ State Key Laboratory of Molecular Reaction Dynamics Dalian Institute of Chemical Physics Chinese Academy of Sciences Dalian Liaoning 116023 P. R. China

**Keywords:** BiVO_4_, charge separation, crystal orientation, photoanode, water oxidation

## Abstract

Preparation of planar bismuth vanadate (BiVO_4_, BVO) photoanode has inspired continuous interest due to its simple preparation and easy scalability, but its efficiency is still retarded by the poor bulk charge separation. Herein, BVO photoanode is successfully prepared with a preferential [010] orientation (denoted as [010]‐BVO) grown on fluorine‐doped tin oxide (FTO) through one simple pyrolysis method for the first time, which exhibits over 21 times of higher photocurrent density of water oxidation with respect to the randomly orientated BVO (denoted as R‐BVO). Strikingly, the orientated BVO photoanode shows an exceptional charge separation efficiency of 97.5% at 1.23 V versus the reference hydrogen electrode (RHE) and a theoretical applied bias photon‐to‐current efficiency (ABPE) value of 2.1% at 0.67 V versus RHE. It is unraveled that the significantly increased carrier transport and collection along the [010] direction should be mainly responsible for the unexpectedly efficient charge separation as well as photoelectrochemical performance. The development of orientated growth planar BVO photoanode may pave the way for designing high‐performance photoanodes for large‐scale solar‐to‐chemical conversion.

## Graphical abstract

The BiVO_4_ photoanode with [010]‐orientated growth is first prepared by simple metal–organic decomposition, which strikingly exhibits by far superior bulk charge separation as well as water oxidation with respect to the randomly BiVO_4_, showing benchmarking photocurrent density and charge separation efficiency of 97.5% at 1.23 V versus.RHE.

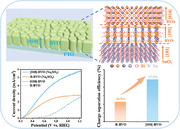

## 1 Introduction

Monoclinic bismuth vanadate (BiVO_4_, BVO) photoanode has been widely investigated for promising water oxidation due to its suitable band gap of 2.4 eV, favorable band‐edge positions, and good photochemical stability.^[^ ^1^ ^]^ To date, both two‐step electrodeposition (ED) and one‐step metal–organic decomposition (MOD) have been mainly employed to prepare it.^[^ ^2^ ^]^ Meanwhile, the feature of the BVO prepared by the two steps of ED is of nanoporous structure to shorten electron transfer distance for favorable charge transport and to provide abundant active sites.^[^ ^3^ ^]^ Consequently, many excellent water oxidation performances on them have been reported.^[^ ^4^ ^]^ However, the ED processes are complicated and cost‐effective, unfavorable for large‐scale preparation. Comparatively, the process of one‐step MOD methodology is relatively simple and easy for large‐scale application,^[^ ^5^ ^]^ but this planar structure leads to a serious bulk charge recombination.^[^ ^6^ ^]^ Consequently, the performances of BVO photoanodes prepared by MOD are generally far inferior to those prepared by the ED process.^[^ ^7^ ^]^ Although our recent work has demonstrated that bulk charge separation of the planar BVO photoanode can be effectively improved by eliminating tetragonal phase generation during pyrolysis,^[^ ^8^ ^]^ there is still a great deal of improvement space for the bulk charge separation.

The regulation of crystal orientation has been developed as an effective strategy to improve the bulk charge separation as well as solar energy conversion efficiency of many semiconductor materials in fields such as photocatalysis^[^ ^9^ ^]^ and solar cells.^[^ ^10^ ^]^ For example, the different surface structures and electronic properties of (010) and (011) facets in the BVO particles have been demonstrated to cause remarkably different photocatalytic properties, in which the (010) facet exhibits more effective electron mobility and water adsorption with respect to the (011) facet.^[^ ^11^ ^]^ This demonstrates that controlling the crystal orientation of BVO may be a promising strategy for improving its property. To date, however, the orientation growth of BVO has been mainly discussed for the powder nanoparticles that can be prepared by hydrothermal method,^[^ ^11^, ^12^ ^]^ Laser ablation (LA) method,^[^ ^13^ ^]^ Langmuir‐Blodgett assembly process, and so on,^[^ ^14^ ^]^ some of them involve complex and expensive equipment, costly target materials, and high operational costs, and are not conducive to large‐area preparation. To the best of our knowledge, the preparation of BVO photoanode with [010]‐orientated grown has not been reported regardless of the ED and MOD method.

**Figure 1:**
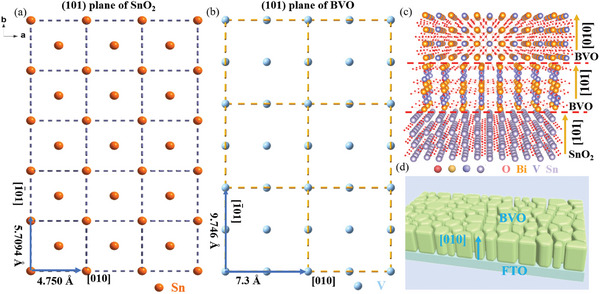
Crystallographic orientation analysis of the [010]‐BVO photoanode: a) Schematic diagram of dimensional matching of cations between the (101) plane of SnO_2_ and b) the (101) plane of tetragonal zircon BVO; c) Atomistic model of hetero‐epitaxial alignment of first [101] orientation growth and subsequent [101] orientation of BVO on SnO_2_ [101]; d) Growth diagram of BVO along [010] direction on the surface of FTO.

Herein, we introduce the first preparation of BVO photoanode with a [010] crystallographic orientation by the simple MOD method, and its success is mainly ascribed to our rational design and controllable synthesis based on the similarity of atomic arrangement between different BVO crystal faces and the exposed crystal faces of SnO_2_ on FTO in Figures S1–S3 (Supporting Information). Theoretical studies demonstrated that the [010] direction of BVO exhibits superior charge transport properties, and the lattice mismatch is a determinant for the oriented growth of BVO film,^[^ ^13^, ^15^ ^]^ based on which the lattice mismatch of facets between support substrate of SnO_2_ and BVO was calculated preferentially in this work. The details and results calculated are given in Table S1 (Supporting Information), based on which between the (101) of tetragonal zircon phase BVO and the (101) plane of SnO_2_ on FTO support is found to exhibit the smallest lattice mismatch, which suggests that the epitaxially matched growth is prone to occur at these interfaces. **Figure** **1**a,b shows the schematic diagram of dimensional matching the planes of SnO_2_ (101) and tetragonal zircon BVO (101), respectively. However, the tetragonal zircon phase was found unfavorable for charge separation.^[^ ^8^ ^]^ Given the similarity in atomic arrangement between the (101) and (010) crystal planes of tetragonal and monoclinic BVO (Figure S3, Supporting Information), a separated deposition was adopted to achieve the first orientation growth of BVO by virtue of the tetragonal phase and subsequent elimination of tetragonal phase, the atomistic model of BVO on SnO_2_ interface is depicted in Figure 1c. Consequently, the pure monoclinic BVO epitaxially grows along [010] direction on FTO can be designed, and the growth diagram is depicted in Figure 1d.

## 2 Result and Discussion

**Figure 2:**
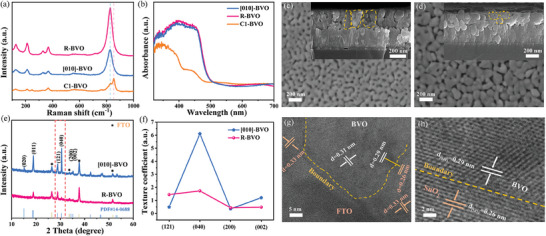
Structural characterizations of typical samples: a) Raman spectra; b) UV–vis absorption spectra; c) SEM image of [010]‐BVO (insert: corresponding cross‐sectional image); d) SEM image of R‐BVO (insert: corresponding cross‐sectional image); e) XRD patterns of [010]‐BVO and R‐BVO; f) Texture coefficient analysis result of [010]‐BVO and R‐BVO; g) Cross‐sectional HAADF‐STEM image of [010]‐BVO; h) The enlarged interface region between the [010] facet of BVO and (101) facet of SnO_2_.

Specifically, the stoichiometric Bi/V precursor was initially applied for the first cycle of spin‐coating to obtain the BVO (referred as C1‐BVO) containing the tetragonal phase of BVO. Afterward, a subsequent spin‐coating step using the vanadium‐rich precursor solution was carried out to achieve [010]‐orientated BVO (denoted as [010]‐BVO), as detailed in the experimental section and Figure S4 (Supporting Information). In contrast, the randomly‐orientated BVO, marked as R‐BVO was obtained directly by repeating the second step of spin‐coating process. Raman spectra were first tested to insight into the phase structure of BVO. As given in **Figure** **2**a, the C1‐BVO exhibits the characteristic peak of the tetragonal phase at 854 cm^−1^,^[^ ^8^, ^16^ ^]^ indicating the formation of tetragonal phase BVO in the first step of preparation. It should be noted that the pure monoclinic phase of BVO is formed for the final [010]‐BVO and R‐BVO photoanode, with the peak at 854 cm^−1^ disappeared. The formation of tetragonal BVO in the C1‐BVO can be further confirmed by the UV–vis absorption spectra (Figure 2b), where the typical absorption edge feature of tetragonal BVO located at 430 nm can be obviously observed,^[^ ^8^, ^17^ ^]^ while this feature peak disappears for the R‐BVO and [010]‐BVO samples, further confirming their pure monoclinic BVO structure. In addition, X‐ray diffraction (XRD) of C1‐BVO shows the peak at 18.3° corresponding to (101) plane, which is consistent with tetragonal structure BVO (JCPDS# 14–0133) (Figure S5a, Supporting Information). In contrast, only pure monoclinic BVO is formed for the randomly oriented R‐BVO (Figure S5b–d, Supporting Information). These results well reveal the importance of the tetragonal phase in inducing the orientated growth along the [010] direction, in accordance with our theoretical prediction in Figure 1.

Subsequently, the basic structures of [010]‐BVO and R‐BVO samples were first evaluated by UV–vis spectra (Figure S6, Supporting Information) and Mott−Schottky (M–S) analysis (Figure S7 and Table S2, Supporting Information), based on which their similar light absorption and flat band potentials can be deduced except that the slope of [010]‐BVO is slightly smaller than that of R‐BVO, indicating much higher electron density of [010]‐BVO. The morphology and thickness of the [010]‐BVO and R‐BVO were then evaluated and given in Figure 2c–d, where both of them have similar thicknesses of ≈300 nm. Notably, the [010]‐BVO shows a larger grain size than the R‐BVO in the vertical direction. XRD shows consistent reflections with monoclinic structure BVO (JCPDS# 14–0688), and the [010]‐BVO has a preferred peak strength of (040) (Figure 2e). The calculated texture coefficient (P) values by XRD data indicate that (040) plane of [010]‐BVO is 6.1, much higher than that of other planes or R‐BVO (P = 1.73) (Figure 2f), confirming the predominant [010] orientation for [010]‐BVO photoanode. The [010] growth orientation can be further supported by the atomic arrangement at the cross‐sectional interface of [010]‐BVO and FTO detected by high‐angle annular dark‐field scanning transmission electron microscopy (HAADF‐STEM) using a focused ion beam. The HAADF‐STEM images are given in Figure 2g–h and Figure S8 (Supporting Information), where the [010]‐BVO/FTO interfaces are continuous with the lattice spacings of 0.29 nm and 0.26 nm parallel to the interface, corresponding to the (040) plane of BVO and the (101) plane of SnO_2_, respectively. This observation confirms the epitaxial growth and alignment of the [010]‐BVO on the FTO substrate, indicating a well‐matched crystallographic orientation.

**Figure 3:**
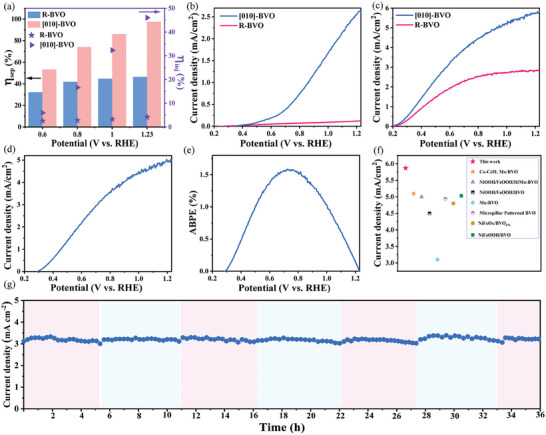
a) Charge transport efficiency (*η*_sep_) and surface charge injection efficiency (η_inj_) of [010]‐BVO and R‐BVO photoanodes; b) Current‐voltage (*J*–*V*) curves of the [010]‐BVO and R‐BVO photoanodes in 0.5 m KBi solution; c) *J*–*V* curves of the [010]‐BVO and R‐BVO photoanodes in 0.2 m Na_2_SO_3_‐containing aqueous solution; d) *J*–*V* curves of PEC water oxidation and e) ABPE of NiFeO_x_/Co_3_O_4_/[010]‐BVO; f) Comparison of photocurrent density of BiVO_4_ photoanodes modified by MOD method in sodium sulfite containing solution; g) Stability test of [010]‐BVO in 0.5 m KBi with 0.2 m Na_2_SO_3_ containing solution at 0.6 V versus RHE.

Unexpectedly, the [010]‐BVO exhibits remarkably superior charge separation and PEC performance to the R‐BVO sample. By referring to the previous publication,^[^ ^18^ ^]^ the surface charge injection efficiency (η_inj_) and charge transport efficiency (η_sep_) within the material were coarsely evaluated by using Na_2_SO_3_ as hole scavenger to assume the surface charge injection efficiency as 100%. Based on their similar light absorption (Figure S6, Supporting Information), the integrated photocurrent density is 6.02 and 6.08 mA cm^−2^ for [010]‐BVO and R‐BVO between 300 and 505 nm, respectively. As given in **Figure** **3**a, on the basis, the calculated η_sep_ values at 1.23 V can reach as high as 97.5% for the [010]‐BVO photoanode, by far higher than that of R‐BVO (46.5%). Similar promotion on the η_inj_ values can be observed. These results indicate that the [010]‐orientated BVO possesses superior charge transport and injection efficiency compared to the random BVO, demonstrating a significant promotion effect of the [010] orientation growth on both bulk charge transfer and surface catalysis. Consequently, the photocurrent density of [010]‐BVO is over 21 times higher than that of R‐BVO shown in Figure 3b. Interestingly, the photocurrent density of [010]‐BVO at 1.23 V versus RHE can reach as high as 5.87 mA cm^−2^ in the sulfite‐containing aqueous solution (Figure 3c), based on which the theoretical applied bias photon‐to‐current efficiency (ABPE) value can be evaluated to reach 2.1% at 0.67 V versus RHE (Figure S9, Supporting Information). Additionally, the absorbed photon‐to‐current conversion efficiency (APCE) of the [010]‐BVO was determined to be 43.4%, which is higher than that of R‐BVO (1.94%) in Figure S10 (Supporting Information). Based on our coarse loading of cocatalyst, the photocurrent density of water oxidation over the NiFeO_x_/Co_3_O_4_/[010]‐BVO can reach 5.02 mA cm^−2^ and the calculated ABPE reaches 1.55% at 0.69 V versus RHE (Figure 3d–e). EDS element distribution of Co_3_O_4_/[010]‐BVO and the XPS analysis of NiFeO_x_/Co_3_O_4_/[010]‐BVO in Figures S11 and S12 (Supporting Information) proved the successful modification of Co_3_O_4_ and NiFeO_x_. To the best of our knowledge, they should be the recording values for water oxidation as well as sulfite‐containing photocurrent density among the BVO photoanodes obtained by MOD (Figure 3f; Table S3 Supporting Information).^[^ ^2^, ^5^, ^6^, ^8^, ^19^ ^]^ It should be noted that the as‐obtained BVO photoanode can also maintain more than 90% current density after irradiation of 36 hours (Figure 3g). The structural and morphological characterizations after stability using XRD and SEM are shown in Figure S13 (Supporting Information). The XRD results confirm that the films maintained their monoclinic phase structure with [010] orientation even after the prolonged stability test. Moreover, the SEM images reveal that the morphology of the films exhibited negligible changes, demonstrating the robust stability of [010]‐BVO films under extended exposure. These results integrally demonstrate the great potential of [010]‐BVO in solar water oxidation as well as solar‐to‐chemical energy conversion.

**Figure 4:**
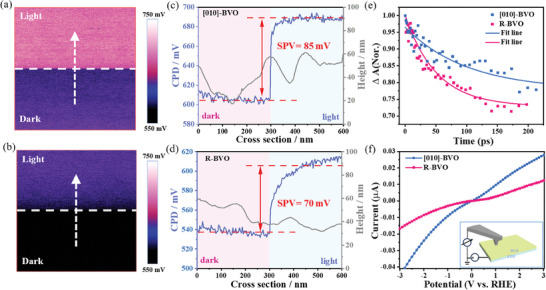
SPV spectroscopy, and corresponding topography height (gray line) and linear distribution of SPV (blue line) values for a,c) [010]‐BVO and b,d) R‐BVO (SPV profiles were calculated by CPD_light_–CPD_dark_); e) TAS kinetics of photogenerated carrier; f) Representative *I*–*V* characteristic curves of [010]‐BVO and R‐BVO (Insert: Illustration on the schematic setup of the C‐AFM measurements).

Surface photovoltage(SPV), defined as the change in the contact potential difference (ΔCPD) under dark and light conditions, was conducted to further insight into the charge separation characteristic of the BVO films.^[^ ^20^ ^]^ **Figure** **4**a–d presents the SPV measurements of [010]‐BVO and R‐BVO. A line scan along the dashed arrow across the BVO particles was plotted to quantitatively identify the CPD difference before and after light excitation. As shown in Figure 4c,d, the [010]‐BVO shows a larger surface photovoltage (85 mV) between illumination and dark and a faster photo‐response with respect to the R‐BVO (70 mV), further confirming the advantage of [010] orientation in promoting charge separation. In addition, CPD (in the same region) of [010]‐BVO was also tested by continuously chopped test to exhibit obvious photo‐response of each section (Figures S14 and S15, Supporting Information), indicating its rapid separation of electron‐hole pairs. Such an enhanced SPV indicates that the [010]‐ BVO is beneficial for the separation of photocarriers, which can be further evidenced by the open‐circuit potentials (OCPs) given in Figure S16 (Supporting Information). The larger difference of OCPs under dark and irradiation conditions of [010]‐BVO corresponds to a more intense band bending. The promoted charge separation observed for the [010]‐BVO can be further confirmed by electrochemical impedance spectroscopy (EIS) and intensity‐modulated photocurrent spectroscopy (IMPS) measurements, based on which the charge transport resistance of [010]‐BVO is much smaller than that of R‐BVO (Figure S17, Supporting Information), and the intercept for [010]‐BVO photoanodes is higher than R‐BVO at a given potential (Figure S18, Supporting Information), revealing that the crystallographic orientations do lower the energy barrier at the crystal–crystal interface as well as facilitated charge collection.

The charge separation properties of [010]‐BVO and R‐BVO were further investigated by carrier dynamics using femtosecond transient absorption spectroscopy (fs‐TAS) as described in Figure S19 (Supporting Information),^[^ ^21^ ^]^ and the result of fitting their kinetics processes by multiexponential functions are shown in Figure 4e and Table S4 (Supporting Information). The significant lifetime extension from R‐BVO (46.5 ps) to [010]‐BVO (100.6 ps) indicates that bulk carrier recombination of [010]‐BVO is effectively inhibited. In addition, the transient photovoltage technique (TPV) was used to characterize the photogenerated carrier dynamics in solution, based on which the photovoltage attenuation of [010]‐BVO shows much slower photovoltage decay than that of R‐BVO (Figure S20, Supporting Information), indicating that [010]‐BVO has fewer recombination centers and better charge separation. On the other hand, the electrical conductivities of [010]‐BVO and R‐BVO photoanodes were measured by the *I*–*V* curves and the C‐AFM technique. As shown in Figure 4f, the current of [010]‐BVO at a constant potential under illumination is higher than that of R‐BVO, suggesting a more robust electron transport capability of [010]‐BVO. Transient photocurrent decay (TPC) in Figure S21 (Supporting Information) shows a similar result that the [010]‐BVO exhibits a higher transient extraction current, and the integrated survived charges is ≈4.2 times of that of R‐BVO, indicating that photo‐induced electrons in the orientation growth [010]‐BVO are rapidly collected on the FTO substrate. All the above results can be well responsible for the observation of superior charge separation performance in the [010]‐BVO photoanode with respect to the R‐BVO.

## 3 Conclusion

In summary, we have successfully synthesized planar BVO photoanodes with a preferential [010] orientation through the simple and low‐cost MOD method for the first time based on the preferential theoretical prediction that takes the lattice mismatches between facets of BVO and SnO_2_ of the FTO support into account. It is demonstrated that the orientated [010]‐BVO exhibits an obvious advantage in the charge transport as well as water oxidation with respect to the randomly dispersed R‐BVO photoanode. Its excellent carrier mobility, conductivity, and increased carrier lifetime along the [010] direction compared to the randomly orientated BVO were detected to be well responsible for its significantly improved charge separation efficiency. It should be noted that the charge separation efficiency of orientated [010]‐BVO unexpectedly reaches as high as 97.5% at 1.23 versus RHE accompanied with theoretical ABPE of 2.1%, which should be the best among the planar BVO photoanodes reported by MOD. Our results should be encouraging to develop large‐scale of planar BVO photoanodes for industrial application of solar energy conversion.

## Conflict of Interest

The authors declare no conflict of interest.

## Supporting information



Supporting Information

## Data Availability

Research data are not shared.
