# Supplementary material for: Preparation and Insight of [010]‐Orientated BiVO4 Planar Photoanode via One‐Step Pyrolysis for Significantly Promoted Charge Separation and Water Oxidation
Source: Adv Sci (Weinh). 2025 Apr 2;12(22):2416474. doi: 10.1002/advs.202416474 (PMC12165083; doi:10.1002/advs.202416474)
Supplement: Supplementary file 1 — Supporting Information [file ADVS-12-2416474-s001.docx]

**Preparation and Insight of [010]-Orientated BiVO_4_ Planar Photoanode via One-Step Pyrolysis for Significantly Promoted Charge Separation and Water Oxidation**

Sainan Zhang^[+][a][b]^, Jiaming Zhang^[+][c]^, Nengcong Yang^[+][b]^,Yejun Xiao^[d]^, Rong Wang^[b]^, Yunfeng Bao^[b]^ Guijun Ma^[c]^, Shengye Jin^[d]^, Fuxiang Zhang*^[a][b]^

[a] S. N. Zhang, Prof. Dr. F. X. Zhang
Department of Chemical Physics, University of Science and Technology of China, Hefei 230026, Anhui, China

E-mail: fxzhang@dicp.ac.cn

[b] S. N. Zhang, Dr. N. C. Yang, Dr. R. Wang, Dr. Y. F. Bao, Prof. Dr. F. X. Zhang
State Key Laboratory of Catalysis, Dalian Institute of Chemical Physics, Chinese Academy of Sciences, Dalian National Laboratory for Clean Energy, Dalian 116023, Liaoning, China

[c] J. M. Zhang, Prof. Dr. G. J. Ma
School of Physical Science and Technology, ShanghaiTech University, Shanghai 201210, PR China

[d] Dr. Y. J. Xiao, Prof. Dr. S. Y. Jin
State Key Laboratory of Molecular Reaction Dynamics, Dalian Institute of Chemical Physics, Chinese Academy of Sciences, Dalian 116023, Liaoning, China

[+] These authors equally contribute to this work

**KEYWORDS**: Water Oxidation, Photoanode, BiVO_4_, Crystal orientation, Charge Separation

1. **Experimental Section**
   1. **Materials**

FTO coated glass (TEC-15 14 Ω/sq, 2 cm × 2 cm), Bi(NO_3_)_3_·5H_2_O (Sinopharm Chemical Reagent, AR), vanadium (IV)-oxy acetylacetonate (Alfa Aesar, 99.8%), citric acid (Sinopharm Chemical Reagent, AR), HNO_3_ (Tianjin Kemiou Chemical Reagent, AR), DMSO (Sinopharm Chemical Reagent, AR), KOH (Sinopharm Chemical Reagent, AR), H_3_BO_3_ (Sigma-Aldrich, 99.5%), FeSO_4_·7H_2_O (Sinopharm Chemical Reagent, AR), NiSO_4_•6H_2_O (Sinopharm Chemical Reagent, AR)，NH_3_·H_2_O (Tianjin Kemiou Chemical Reagent, AR), ethanol (Sinopharm Chemical Reagent, AR) cobalt acetate (Sinopharm Chemical Reagent, AR).

- 1. **Preparation of BiVO_4_ film**

First, 0.6 mmol of Bi(NO_3_)_3_⋅5H_2_O was dissolved in 1mL of dimethyl sulfoxide, and 0.6 mmol of VO(acac)_2_ was added after 20 min of ultrasonic treatment. The BiVO_4_ (BVO) precursor solutions were then spin coated on the FTO substrate at 3500 rpm for 30 s. After the spin coating, the samples were immediately annealed on muffle furnace at 500℃ for 2 h, the obtained BVO during one cycle spin-coating referred as C1-BVO. The repeated spin-coating and annealing processes were performed using a 10% excessive concentrations of VO(acac)_2_ precursor solution to achieve [010]-oriented BVO (denoted as [010]-BVO). In contrast, the randomly-oriented BVO was obtained directly by repeating the second spin-coating process using the vanadium-rich precursor solution several times, and finally heated in muffle for the same time marked as R-BVO, the BVO obtained by one cycle spin-coating of R-BVO is marked as R-BVO-1.

**1.3 The loading of cocatalyst**

The preparation of Co_3_O_4_ Colloidal solution：add 0.4 mL NH_3_·H_2_O into the 25 mL ethanol solution of 1mM cobalt acetate, stir for 15min, and then heat it in the oven at 120℃ for 1h. After the reaction, the solution was removed and ultrasonic for 30 min to obtain Co_3_O_4_ colloidal solution. Then BVO was impregnated for 10 min, and then heated in Muffle furnace at 450℃ for 2 h to obtain the Co_3_O_4_/BVO photoanode.

The NiFeO_x_ cocatalyst was deposited using a photo-assisted linear sweep voltammogram (LSV) method under AM 1.5 G illumination.^[1]^ Briefly, a 0.5 M borate buffer solution was prepared by dissolving 0.1 mol of H_3_BO_3_ in 200 mL of Milli-Q water, followed by adding KOH to achieve a pH of 8.3. Then, 20 mg of FeSO_4_·7H_2_O and 2 mg of NiSO_4_·6H_2_O were dissolved in the above borate buffer solution previously purged with N_2_ for 20 min. Under AM 1.5 G illumination from the FTO side, LSV was performed from -0.3 to 0.5 V versus Ag/AgCl with a scan rate of 20 mV s^–1^ for 6−7 circle. The obtained samples were denoted as NiFeO_x_/Co_3_O_4_/[010]-BVO.

**2. Material characterization**

The ultraviolet−visible absorption spectra (UV-Vis) from 350 to 700 nm were taken on Shimadzu-UV2600 spectrophotometer. Powder X-Ray diffractions (XRD) were measured on a Rigaku SmartLab powder diffractometer operating at 40 kV and 200 mA with Cu Kα radiation (λ = 0.154 nm). The morphologies of samples were imaged by a scanning electron microscope FESEM (JSM-7900, JEOL). The elements of the samples were identified by the equipped energy dispersive X-ray spectroscopy (EDX). The TEM image and SAED pattern were obtained using a JEOL transmission electron microscope (JEM F200) at an accelerating voltage of 200 kV.

The spatially-resolved surface photovoltage microscope (SPVM) setup was installed in a Kelvin probe force microscopy (KPFM) setup (Bruker Dimension FastScan) consisting of a modified Dimension Icon. Samples were prepared by transferring the particles onto a carbon conductive adhesive. ^1^ KPFM measurements were conducted in lift mode, where the topography and surface potential signals were acquired sequentially. For surface photovoltage (SPV) measurements, the lift mode with a height of 100 nm was employed. The contact potential difference (CPD), which represents the surface potential, was measured under ambient conditions in amplitude-modulated mode. ^[3]^ The CPD is defined as:

CPD = φ_sample_− φ_tip_ (S1)

Where φ represents the potential, and the smaller the measured CPD, the lower the sample potential, and the higher the corresponding work function. The Pt/Ir-coated Sb-doped Si tip (SCM-PIT-V2) was used as a Kelvin tip. A 450 nm LED (2 mW cm^-2^) almost parallel to the substrate was used to measure the CPD under illumination. SPV is the change in contact potential difference before and after illumination, the SPV is defined as:

SPV = ∆CPD = CPD_light_-CPD_dark_  (S2)

The femtosecond transient absorption setup is based on a regenerative amplified Ti:sapphire laser from Coherent corporation (800 nm, 35 fs, 6 mJ/pulse, and 1 kHz repetition rate), nonlinear frequency mixing techniques and a transient absorption spectrometer (TA100) from Time-tech Spectra LLC. Briefly, the 800 nm output pulse from the regenerative amplifier was split in two parts with a 50% beam splitter. The transmitted part was used to pump a TOPAS Optical Parametric Amplifier (OPA) which generated a wavelength-tunable laser pulse from 250 nm to 2.5 μm as pump beam. The reflected 800 nm beam was split again into two parts. One part with less than 10% was attenuated with a neutral density filter and focused into a 2 mm thick sapphire or CaF_2_ window to generate a white light continuum (WLC) used for probe beam. The probe beam was focused with an Al parabolic reflector onto the sample. The delay between the pump and probe pulses was controlled by a motorized delay stage. All TA experiments were performed under ambient conditions.

The TAS curves can be well fitted by a multiple-exponential function of time (t):

$A \left( t \right)={A_{0}+ A}_{1}e^{-\frac{t}{\tau_{1}}}+ A_{2}e^{-\frac{t}{\tau_{2}}}$ (S3)

The *τ*_1_ and *τ*_2_ refer to the time constant of the fast and slow decay processes, respectively. All the fitting time parameters are summarized in Table S3.

**3. Photoelectrochemical measurements**

All the photoelectrochemical (PEC) measurements were conducted in a typical three-electrode cell using the electrochemical workstation (Modulab XM, Solartron). A Xe arc lamp equipped with an AM 1.5 G filter was used as the light source and the light intensity was calibrated to ca. 100 mW/cm^2^ with a standard silicon cell detector (Newport). Current-voltage (J-V) curves under irradiation and dark were recorded with a scan rate of 20 mV∙s^-1^. Stability was test at 0.6 V versus reversible hydrogen electrode (RHE), the electrolyte was refreshed after a period of testing during the testing process. Electrochemical impedance spectroscopy (EIS) Nyquist plots were collected with an AC voltage amplitude of 10 mV under AM 1.5 G illumination (frequency range: 0.1 Hz~100 kHz). Photovoltage measurement was performed according to a previous report, open circuit potentials (OCP) of photoanodes were measured in dark and under AM 1.5 G illumination after stabilizing for enough time. The electrolyte solution was 0.5 M potassium borate electrolytes (KBi, pH = 9.5), 0.2 M Na_2_SO_3_ was added to the electrolyte as a hole scavenger. The counter and reference electrodes used were platinum foil and Ag/AgCl (3 M KCl) electrode. The potential was converted to the RHE by the Nernst equation as below: ^[2]^

E_RHE_ = E_(Ag/AgCl)_ + 0.059pH + E^θ^_(Ag/AgCl)_ (S4)

where E_RHE_ refers to the converted potential *versus* RHE. The value of E^θ^_(Ag/AgCl)_ is 0.197 V at ambient temperature (25℃) and E_(Ag/AgCl)_ is the obtained potential versus Ag/AgCl.

Applied bias photon-to-current efficiency (ABPE) can be calculated using the following equation:^[3]^

𝐴𝐵𝑃𝐸 (%) = 𝐽×(1.23− 𝑉_𝑏ias_)/P_total_ (S5)

Where J is the photocurrent density (mA cm^-2^) obtained from the electrochemical workstation. V_bias_ refers to the applied bias versus RHE (V), and P_total_ is the total light intensity of AM 1.5 G (100 mV cm^–2^).

Mott-Schottky (M-S) curves were obtained in the voltage window of 0.5~1.2 V vs. RHE in the dark (increment: 10 mV, frequency: 1 kHz). According to the M-S curves, the flat band potential can be obtained using then following equation:

${C_{SC}}^{-2}=\frac{2}{e\varepsilon_{0}\varepsilon}(V-V_{fb})$ (S6)

The electronic charge (e) is 1.6 × 10^-19^ C, vacuum permittivity (ε_0_) is 8.85 × 10^-12^ F m^-1^, and relative permittivity (ε) is 7 for BiVO_4_. The summarized flat potentials are listed in Table S2.

Intensity-modulated photocurrent spectroscopy (IMPS) was measured in Solartron Modulab XM to identify the several key processes involving charge transfer and surface recombination for PEC water oxidation. In this measurement, a varying frequency of the sinusoidal perturbing signal for light intensity (10 kHz~100 mHz) is superposed on constant illumination (395 nm LED, Thorlabs) at several specific applied potentials, and the periodic photocurrent signal response of the system is collected as a function of the light modulation frequency. The real axis [Re (H)] intercept at medium frequencies is in connection with the charge separation efficiency multiplied by the light harvesting efficiency at a given wavelength (CSE×LHE), while the low frequency intercept on Re (H) can be defined as the external quantum efficiency (EQE).

**4. Calculation**

**4.1 Calculation of the texture coefficient**s **of BVO photoanodes**

The texture coefficient (P) referred to the preferential of the orientations was calculated from the XRD data (Figure 2f) using the following equation:^[4]^

$P_{(hkl)}=\frac{I_{(hkl)}}{\sum I_{(hkl)}} /\frac{{I_{0}}_{(hkl)}}{\sum{I_{0}}_{(hkl)}}$ (S7)

Here, the I_(hkl)_ is the measured peak intensity corresponding to the (hkl) plane, while I_0(hkl)_ is the standard intensity from the JCPDS card of the monoclinic BVO. For a preferred orientation, the *P* for the corresponding plane should be greater than 1. Due to the standard intensity of (121) plane is relatively higher than the other peaks, the *P* of R-BVO is only a litter larger than 1.

**4.2 Transient photovoltage (TPV)**

The fitting photovoltage curve was obtained with subtracting the exponential part of the fitting function:

$V\left( t \right)= V_{0}+A_{1}e^{-(t-t_{0})/\tau_{1}}$ (S8)

**4.3 Steady- state photovoltage**

This photovoltage is derived from the divided spatial configuration of separated carriers in space charge region under steady irradiation, which can be calculated by subtracting the open circuit potential under dark to that under illumination.

**4.4 Calculation of theoretical photocurrent density**

The theoretical photocurrent density (*j*_abs_) is calculated based on the light absorption. To calculate *j*_abs_, light harvest efficiency (LHE) should be first obtained on the basis of its light absorbance (A) as follows (λ is the wavelength):

LHE (λ)=1-T%-R% (S9)

The light absorbance (A) is measured experimentally by transmission and reflection spectroscopy, then, *j*_abs_ can be calculated:^[3]^

*j*_abs_ = ∫ J_flux_(λ)dλ (S10)

$J_{\mathrm{flux}}\left( \lambda\right)=\frac{N_{\mathrm{ph}}\left( \lambda\right)\times LHE\left( \lambda\right)}{N_{A}}\times F\times1000$ (S11)

Herein, J_flux_ is the current flux and N_ph_ is the photon flux; N_A_ is the Avogadro constant, F is the faraday constant. According to Figure S6, they keep the similar light integral area of 80.2% and 81%, thus, the integrated *j*_abs_ was calculated to be 6.02 mA cm^-2^ and 6.08 mA cm^-2^ for the [010]-BVO and R-BVO photoelectrodes.

Basically, the photocurrent density (J) is determined by three fundamental processes: the theoretical current density (J_abs_) according to the light absorption, charge transport efficiency (*η*_sep_) and charge injection efficiency (*η*_inj_) at the interface between the electrode and solution. Since the oxidation kinetics of hole scavengers (e.g. Na_2_SO_3_) is effortless enough, assuming that the amount of surface charge injection efficiency in hole scavenger of Na_2_SO_3_ is 100%. The η_sep_ and η_inj_ can be calculated using the following equations:

𝜂_𝑠𝑒𝑝_= 𝐽_𝑁𝑎2𝑆𝑂3_/𝐽_𝑎𝑏𝑠_ (S12)

𝜂_inj_= 𝐽_water_/𝐽_𝑁𝑎2𝑆𝑂3_  (S13)

Where J_abs_ is the unity converted photocurrent density from the light absorption, while 𝐽_𝑁𝑎2𝑆𝑂3_ is the photocurrent density obtained in 0.5 M KBi (pH 9.5) in the presence of 0.2 M Na_2_SO_3_, and 𝐽_water_ is the photocurrent density for water oxidation in 0.5 M KBi (pH 9.5).

**4.5** **Calculation of the lattice mismatches**

According to the formula:^[2, 5]^

$Lattice mismatches \left( \frac{\alpha_{\mathrm{substrate}}-\alpha_{\mathrm{film}}}{\alpha_{\mathrm{film}}} \right)$ (S14)

Where α represents the lattice constants of the different crystal faces.

**4.6 Calculation of the electron conductivity**

From the current–voltage (I–V) measurement, the conductivity (σ) was calculated by Equation S14:

𝜎 =𝐿/𝑅𝐴 = 𝐿𝐼/𝐴𝑉 (S15)

Where I and V are the current and voltage in I-V curve. The film thicknesses [010]-BVO and R-BVO were 300 nm. The tip area (A) used in the test is 1.9625 × 10^-3^ cm^2^, R is the electric resistance of the film.

**Result and Discussion**

**Calculation of the lattice mismatches**
The structural similarities between different BVO crystal phases and SnO_2_ were analyzed. Rough comparison of lattice symmetry and parameters (BVO (z-t), P1 a = b = 7.3 Å, c = 6.457 Å, α=β=γ= 90°; BVO (s-m), P1, a =5.194 Å, b = 5.094 Å, c =11.697 Å, α=β= γ= 90°; SnO_2_, P4_2_, a = b = 4.738 Å, c = 3.188 Å, α=β=γ= 90°). The atomic arrangement of the SnO_2_ (101) plane，tetragonal zircon (z-t) BVO (101) and monoclinic scheelite (m-s) BVO (010) planes were given and analyzed (Figure S1). Based on the lattice mismatch equation S14, the mismatch between the five-fold-repeated SnO_2_ (101) plane along its [-101] direction (*d*_[-101]SnO2(101)_ = 5.709 Å) and BVO (101) plane along its [-101] direction (*d*_[-101]BVO(101)_= 9.746 Å) and that between three-fold-repeated SnO_2_ (101) plane along its [010] direction (*d*_[010]SnO2(101)_ = 4.753 Å) and two-fold-repeated BVO (101) plane along its [010] direction (*d*_[010]BVO(101)_ = 7.3 Å) are only -2.36% and -2.34%，the calculated lattice mismatches of (010)_BVO_||(101)_Sub_ for [100]_BVO_||[-101]_SnO2_ and [001]_BVO_||[010]_SnO2_ are 9.9 and 1.58, respectively, less than 15%, indicating that the epitaxial growth along SnO_2_ (101) plane can be extended,^[5-6]^ the results of the calculations are shown in Table S1. Moreover, the (101) crystal plane of z-t BVO is less mismatched with that of SnO_2_, indicating that it is easy to extend the epitaxial growth along the SnO_2_ (101) plane_,_ the atomistic model of the z-t-BVO (101) and SnO_2_ (101) interface was displayed in Figure S2. Given the similarity of atomic arrangement between the z-t (101) and m-s (010) crystal planes depicted in Figure S3, with only minor difference in the degree of distortion, we can leverage controlled experimental design through stepwise spin-coating process to firstly prepare z-t BVO on FTO based on our prior work. Subsequently, by adjusting the Bi:V ratio during the spin coating process, the [010]-oriented growth BVO can be obtained on the basis of z-t BVO, while eliminating the existence of tetragonal phase. This manipulation leads to the epitaxial alignment of crystalline BVO with SnO_2_ on the FTO substrate in the [010] direction, with the crystallographic orientations [100]_BVO_||[-101]_Sub_ and [001]_BVO_||[010]_Sub_.


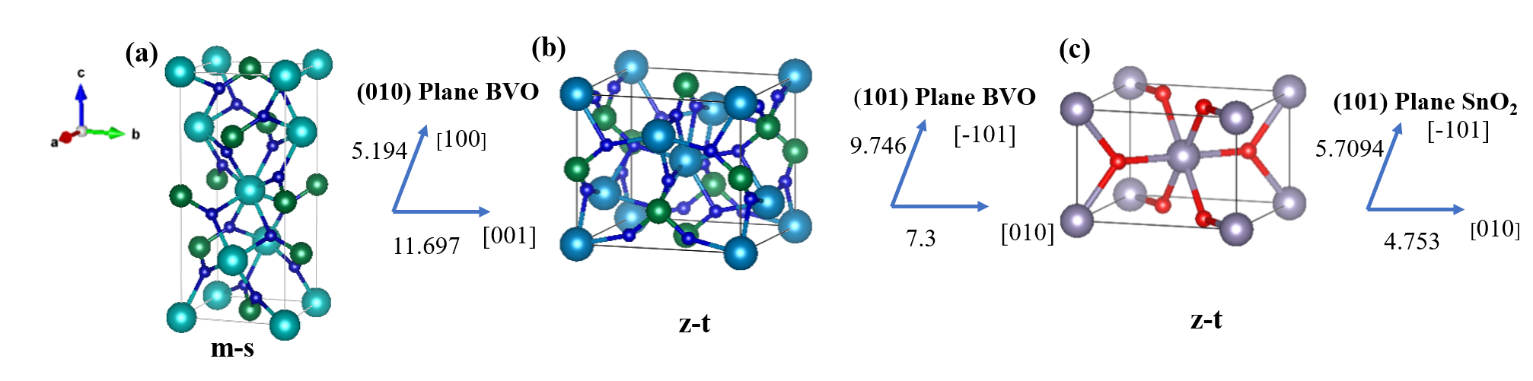
**Figure S1.** (a) Monoclinic scheelite (m-s) unit cell of BVO and atomic arrangement of BVO (010) plane. (b) Tetragonal zircon (z-t) unit cell of BVO and atomic arrangement of BVO (101) plane (c) Tetragonal zircon (z-t) unit cell of rutile SnO_2_ and atomic arrangement of SnO_2_ (101) plane.

**Table S1.** Crystal structures, lattice parameters and lattice mismatch values of z-t BVO, s-m- BVO, and FTO.

| Substrate | Crystal system | a(Å) | b | c | _β (°)_ | [-101]_BVO_\|\|[-101]_Sub_ | [010]_BVO_\|\|[010]_Sub_ | [100]_BVO_\|\|[-101]_Sub_ | [001]_BVO_\|\|[010]_Sub_ |
| --- | --- | --- | --- | --- | --- | --- | --- | --- | --- |
| BVO | z-t | 7.3 | 7.3 | 6.457 | 90 | -2.36 | -2.34 |  |  |
| BVO | m-s | 5.194 | 5.094 | 11.697 | 90 |  |  | 9.9 | 1.58 |
| FTO  (SnO_2_:F) | Tetragonal | 4.738 | 4.738 | 3.188 | 90 |  |  |  |  |

Atomic configurations of tetragonal zircon BVO (101) plane are well-matched (101) plane of SnO_2_.


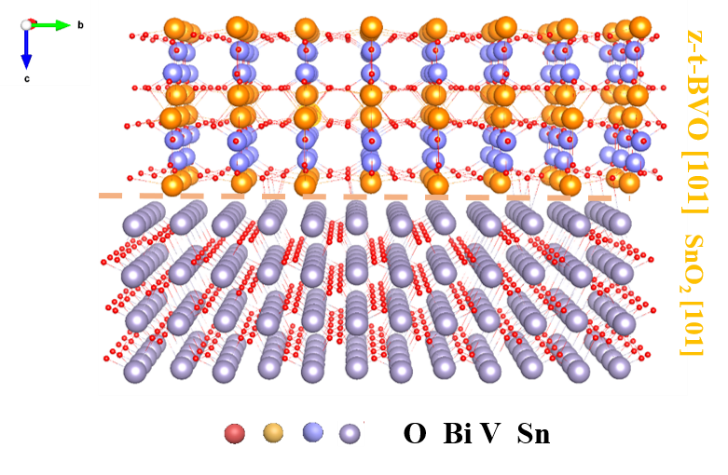


**Figure S2.** (a) Atomistic model of the z-t BVO (101) and SnO_2_ (101) interface.


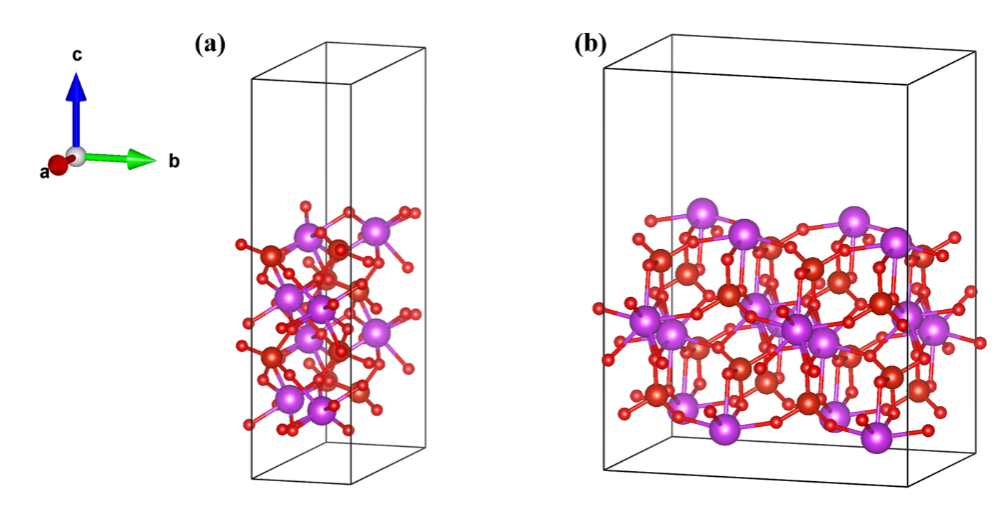


**Figure S3**. Crystal structure of (a) m-s BVO (010) plane and (b) z-t BVO (101) plane.


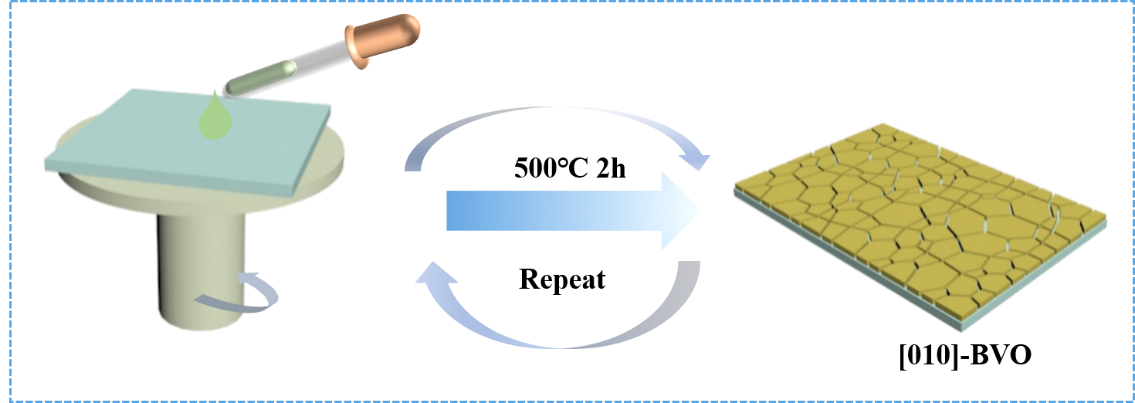


**Figure S4.** Synthesis schematic illustration of [010]-BVO photoanodes.


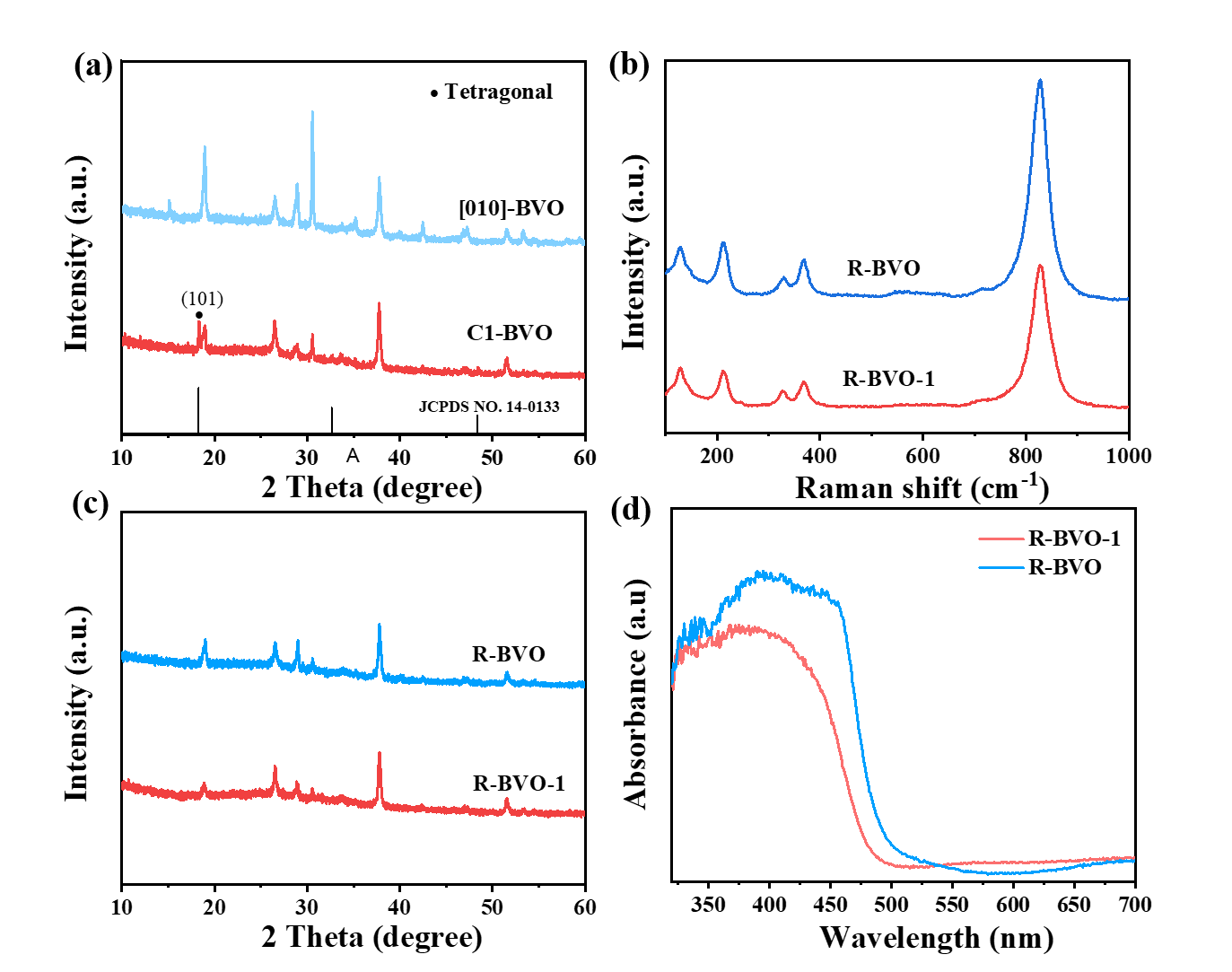


**Figure S5.** (a) XRD of C1-BVO and [010]-BVO; (b-d) Raman, XRD and UV-vis absorption spectra of R-BVO and R-BVO-1 obtained by one cycle spin-coating of R-BVO.


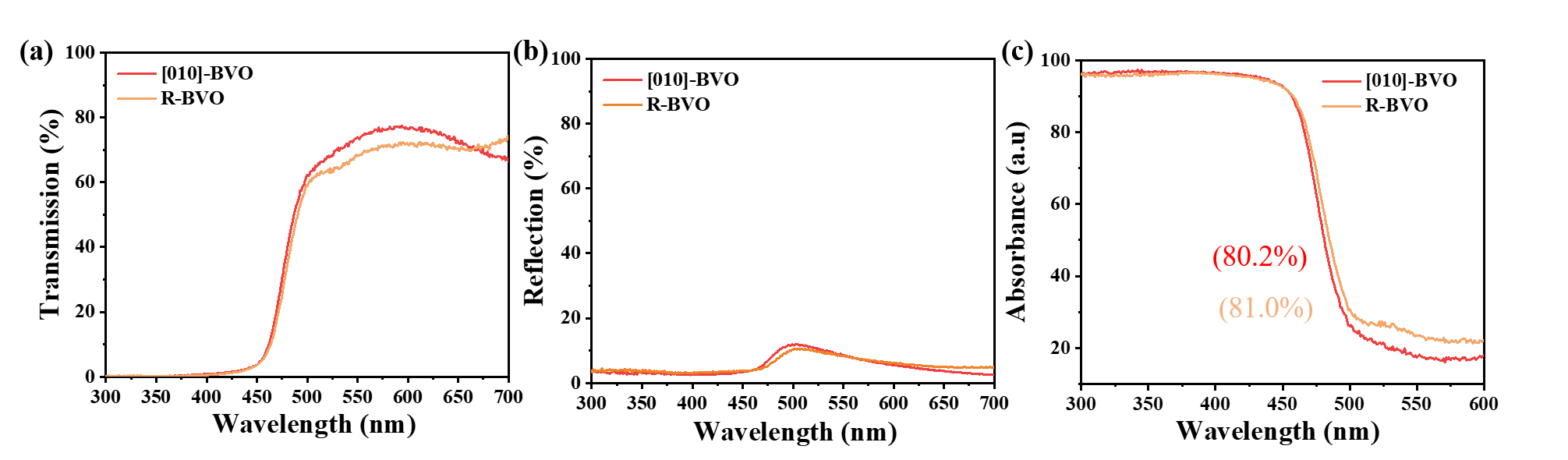
**Figure S6.** Optical properties of [010]-BVO and R-BVO (a) Transmittance spectra (b) Reflection and (c) Uv-vis absorption spectra (100-T%-R%). The integrated values in the wavelength range of 300-505 nm were shown in parentheses.

**Figure S7.** Mott–Schottky plots of of [010]-BVO and R-BVO in KBi electrolyte solution (0.5 M, pH=9.5).

**Table S2**. Flat band potential and donor concentration of [010]-BVO and R-BVO photoanodes

| Sample | Flat band potential Vfb (Vs RHE) | Electron density N_d_ (cm^-3^) |
| --- | --- | --- |
| [010]-BVO | 0.301 | 23.98×10^19^ |
| R-BVO | 0.295 | 13.28×10^19^ |


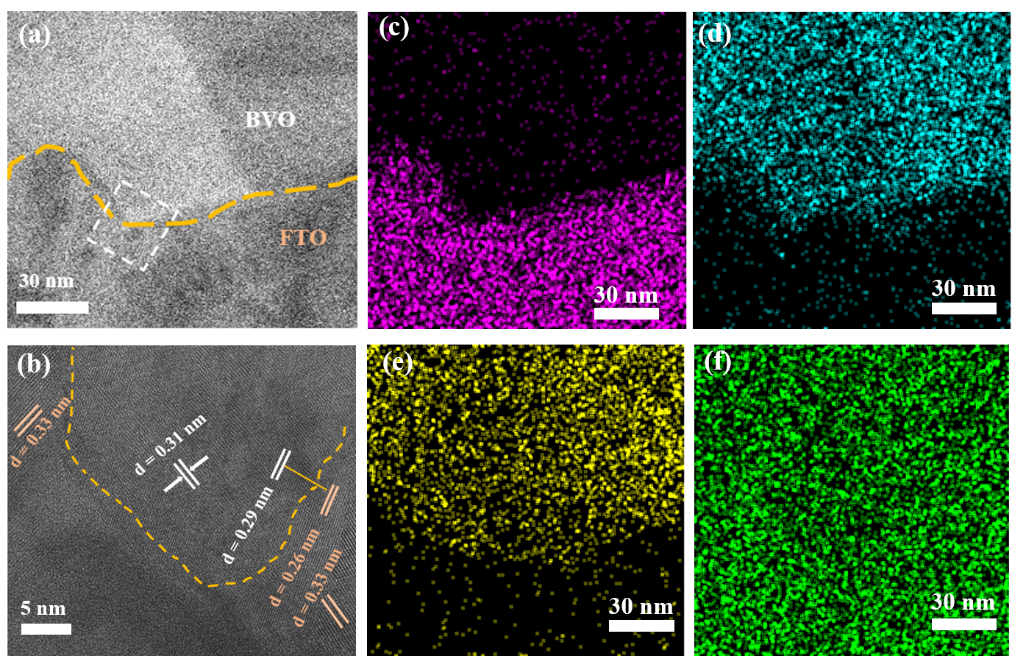


**Figure S8.** Crystallographic orientation analysis of the [010]-BVO photoanode. (a) Cross-sectional HAADF-STEM image of [010]-BVO, (b) Enlarged images extracted from the white rectangles in (a), and the corresponding energy spectrum of (c) Sn, (d) Bi, (e) V, (f) O.


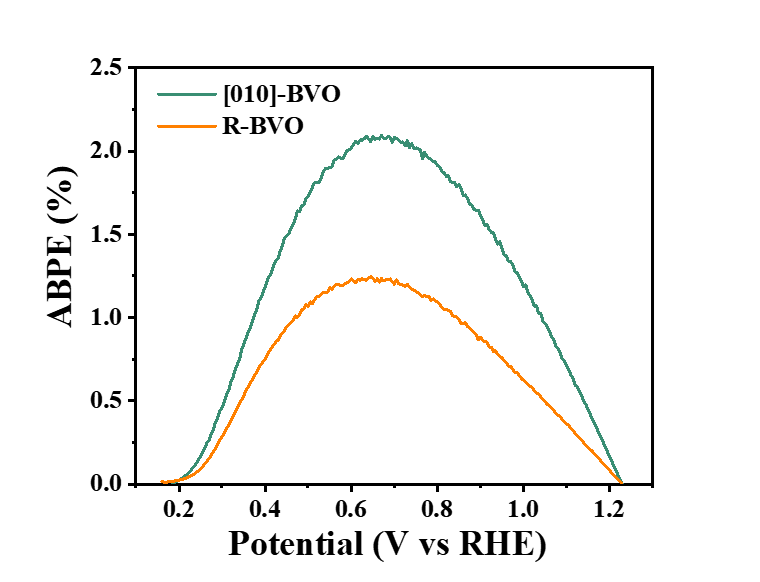


**Figure S9.** The theoretical applied bias photon-to-current efficiency (ABPE) curve of [010]-BVO and R-BVO photoanodes.


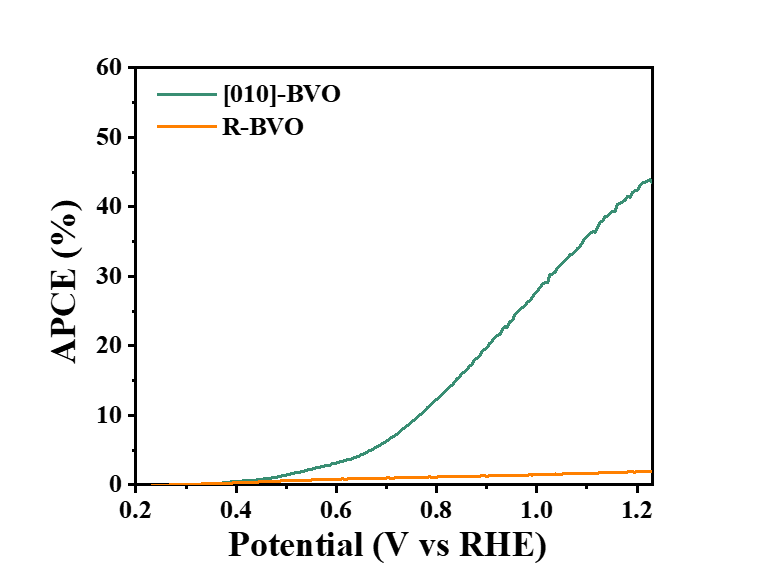


**Figure S10.** The absorbed photon-to-current conversion efficiency (APCE) curve of [010]-BVO and R-BVO photoanodes.


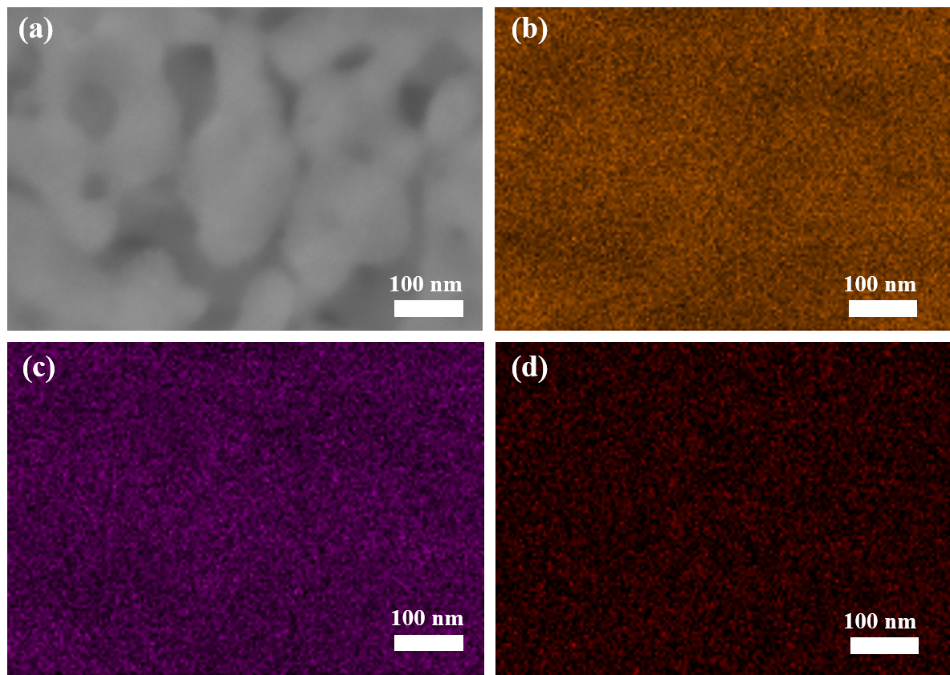


**Figure S11.** (a) Scanning electron microscopy (SEM) of Co_3_O_4_/[010]-BVO and the corresponding energy spectrum of (b) Bi, (c) V, (d) Co.


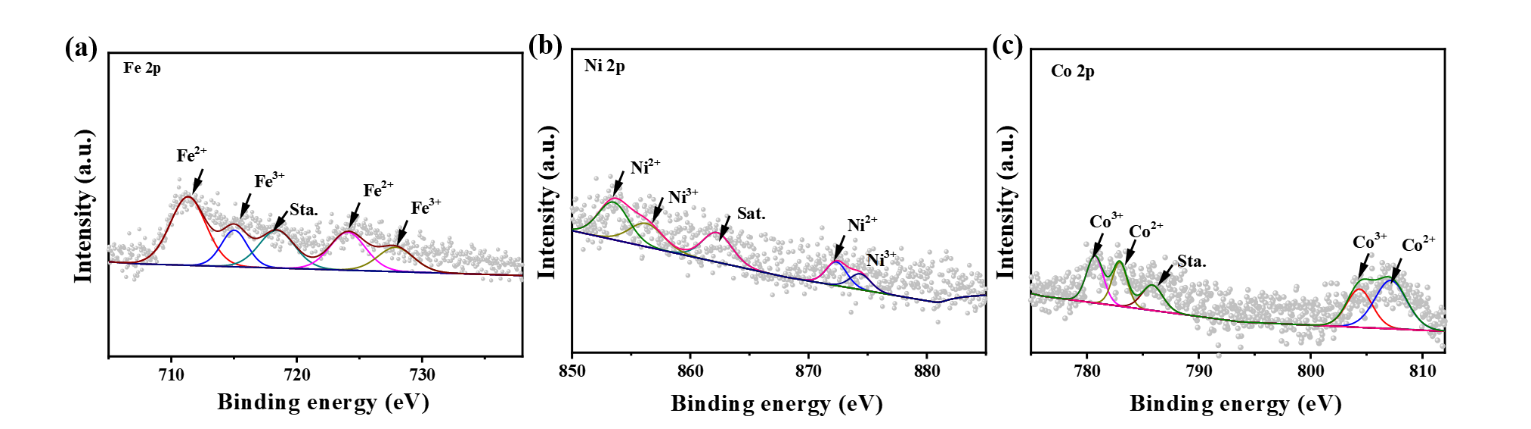


**Figure S12.** XPS spectra of NiFeO_x_/Co_3_O_4_/[010]-BVO: (a) Fe 2p, (b) Ni 2p, (c) Co 2p.


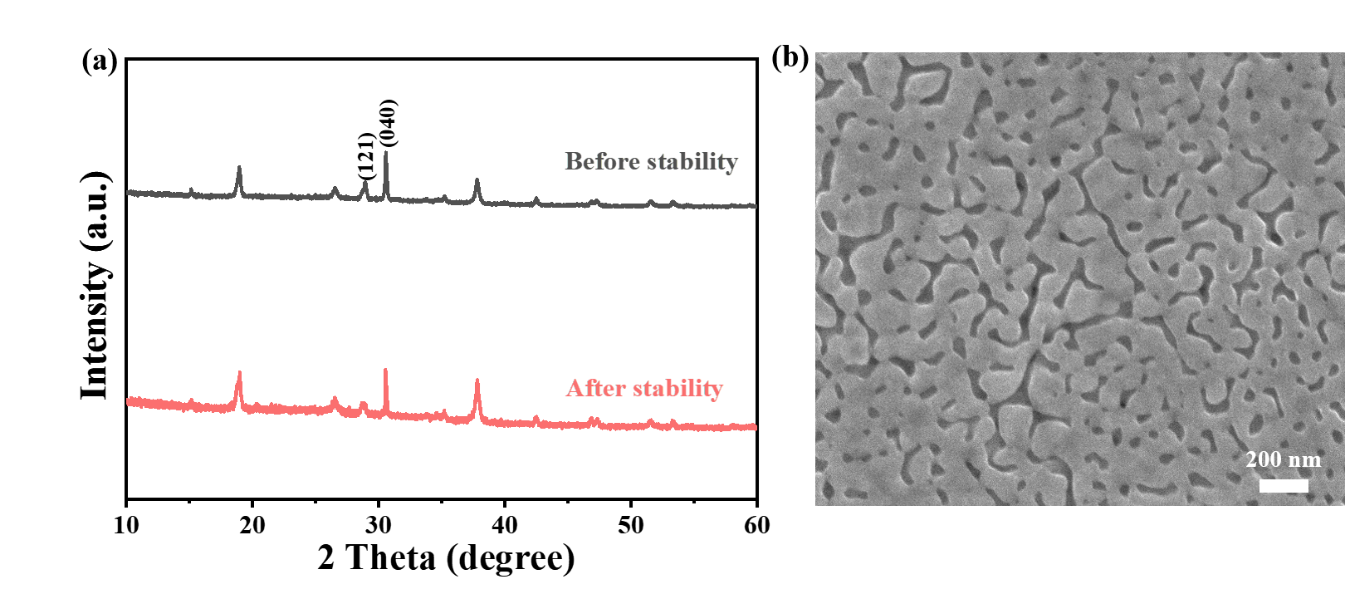


**Figure S13.** (a) XRD of [010]-BVO before and after stability test, (b) SEM of [010]-BVO after stability test.


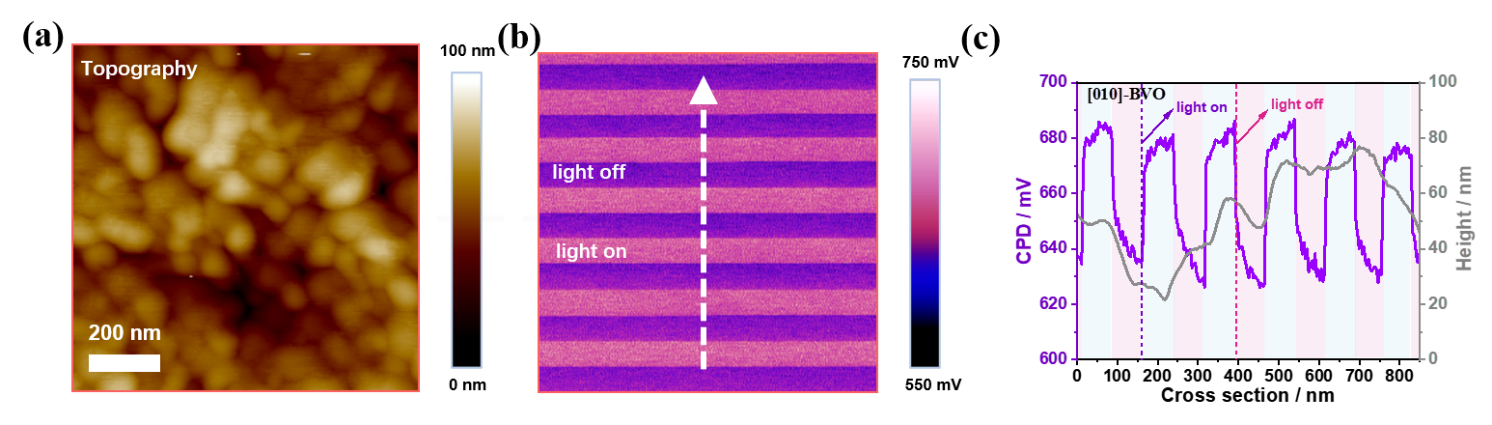


**Figure S14.** (a) AFM topographic images, (b) contact potential difference (CPD) in continuous chopping state and (c) corresponding topography height (gray line) and SPV (purple line) cross-section images of [010]-BVO.


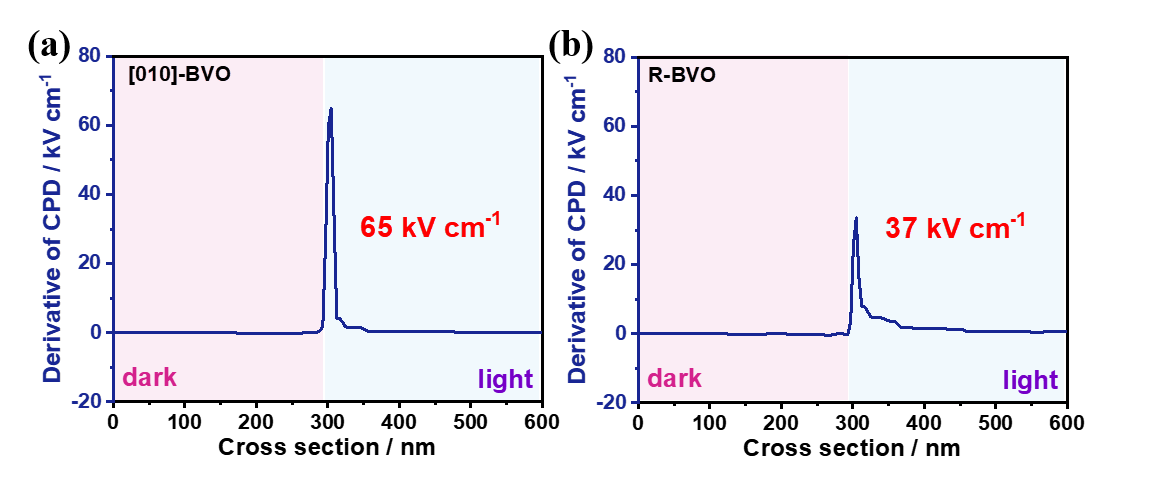


**Figure S15.** The derivative of (a) [010]-BVO and (b) R-BVO from Figure 4 (c) and (d).


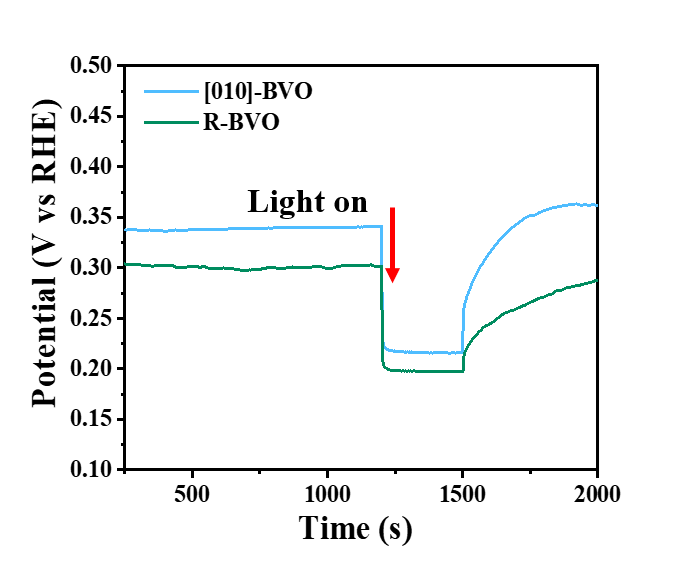


**Figure S16.** Open circuit potentials (OCPs) of [010]-BVO and R-BVO electrodes under dark and AM 1.5G irradiation conditions.


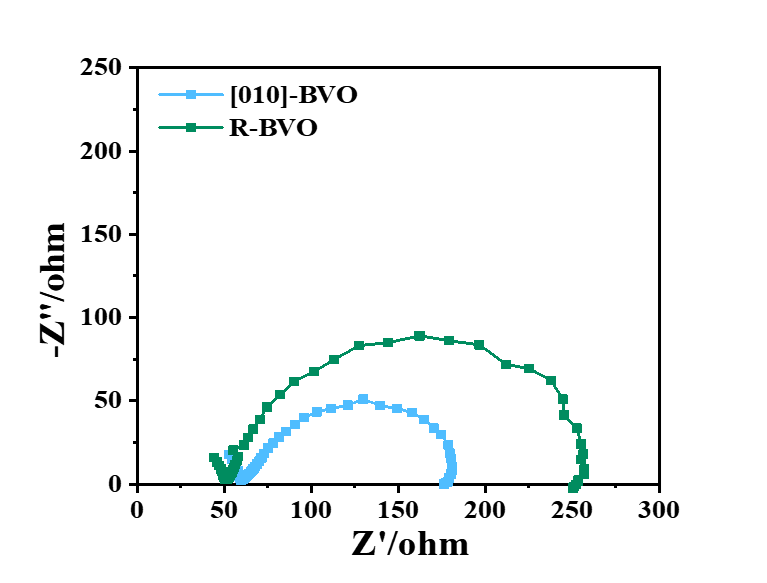


**Figure S17.** Electrochemical impedance spectroscopy (EIS) of [010]-BVO and R-BVO photoanodes.


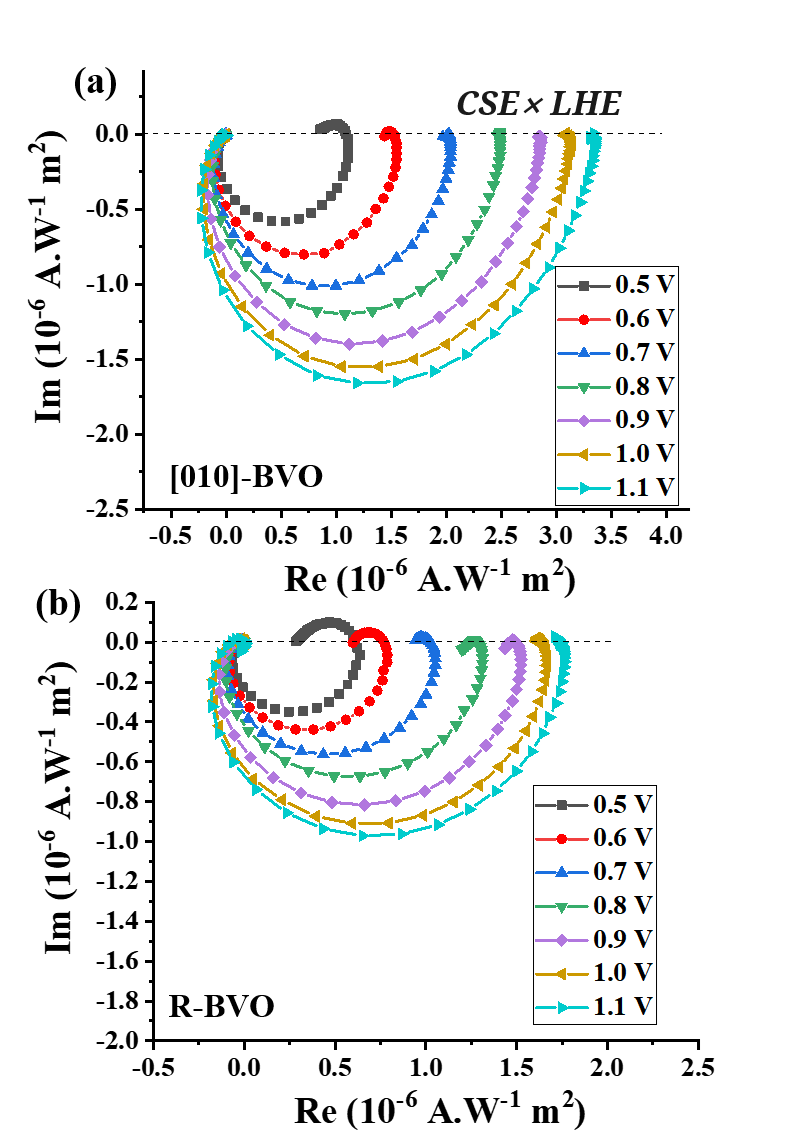


**Figure S18**. Intensity modulated photocurrent spectroscopy (IMPS) of [010]-BVO and R-BVO at different potential vs. RHE.


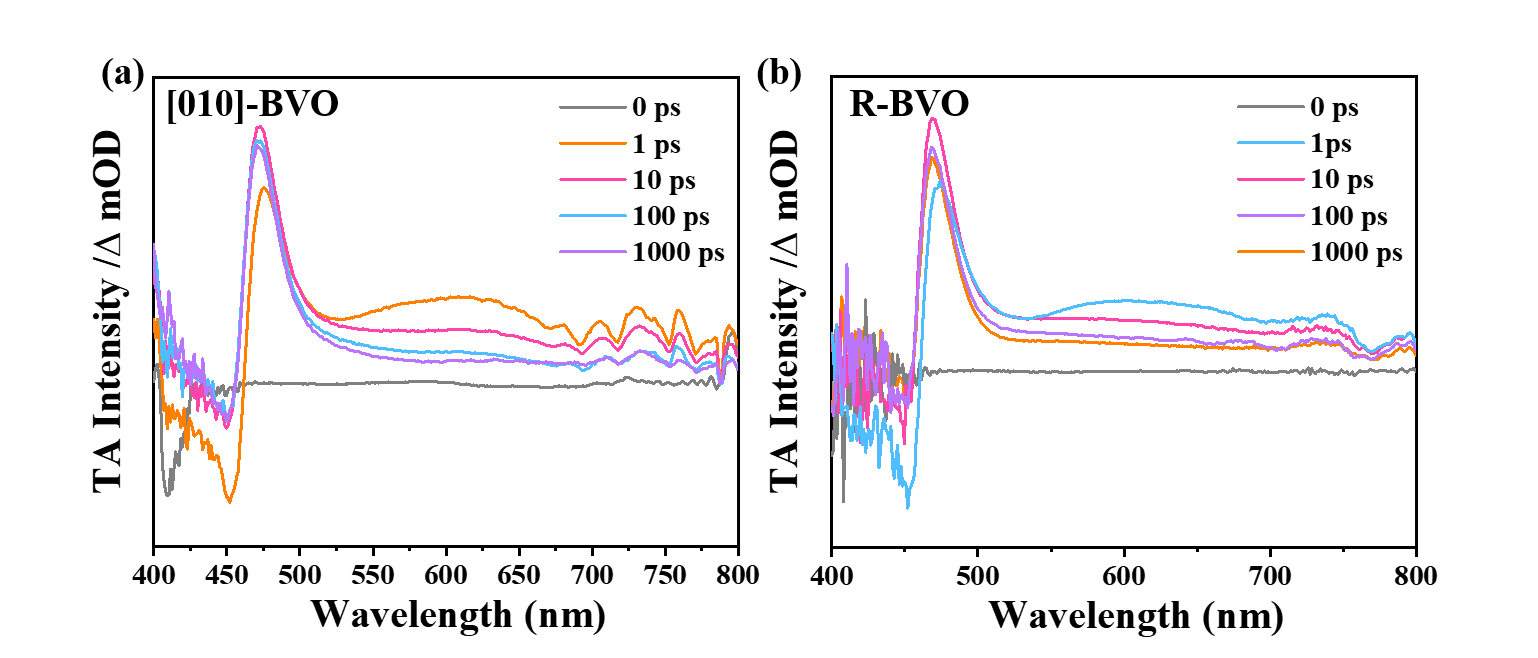


**Figure S19.** Femtosecond transient absorption spectra (fs-TAS) of (a) [010]-BVO and (b) R-BVO.


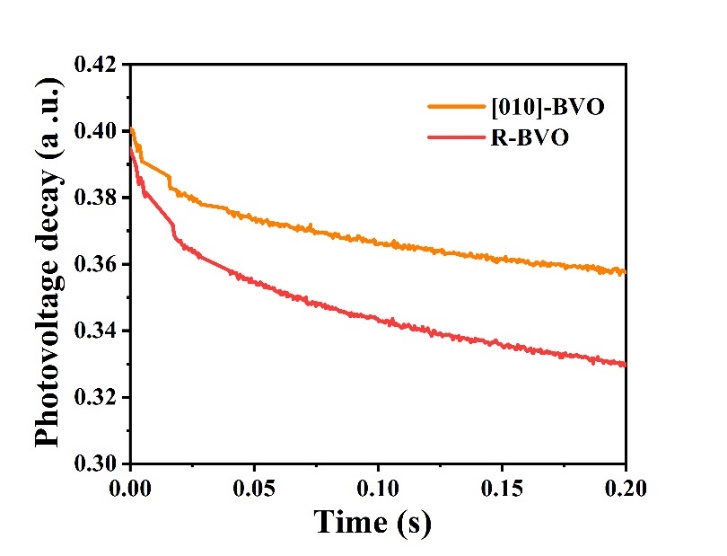


**Figure S20.** Transient photovoltage decay (TPV) of [010]-BVO and R-BVO.


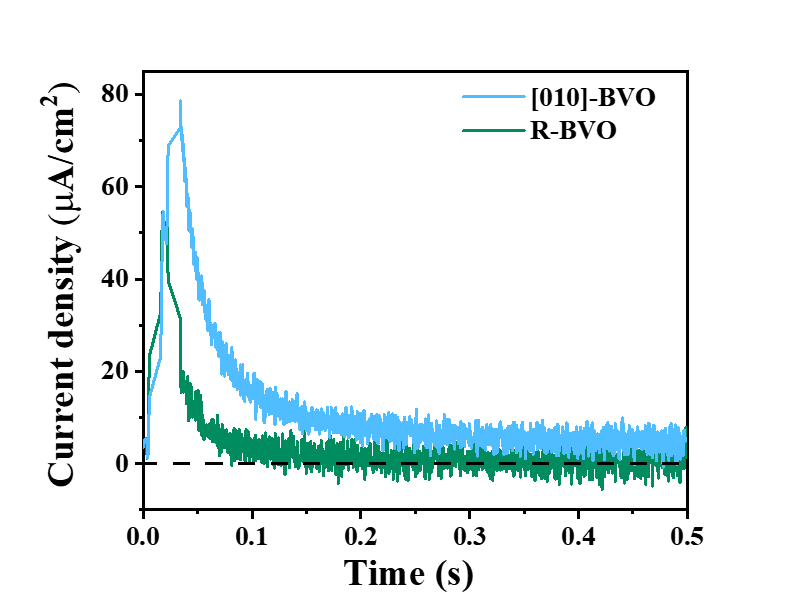


**Figure S21.** Transient photocurrent decay (TPC) for [010]-BVO and R-BVO.

**Table S3** Comparison of photocurrent density between water oxidation and sodium sulfite oxidation for modified BiVO_4_ photoanodes obtained by MOD method

| Improvement strategy | Photoanode | Na_2_SO_3_ (mA cm^-2^) | OER (mA cm^-2^) | η_transport_ (%) | Ref. |
| --- | --- | --- | --- | --- | --- |
| [010]-Oriented growth | BVO | 5.87 | 5.03 | 97.5 | This work |
| NaBH_4_-treated and Mo-doped | NiOOH/FeOOH/H/ Mo:BVO | 5.0 | 5 | -- | ^[7]^ |
| Solvent and Bi:V ratios | NiFeOOH/BiVO_4_ | 5.03 | 4.53 |  | ^[8]^ |
| Mo-doped | Mo: BiVO_4_ | 3.1 | 1.8 | 79 | ^[9]^ |
| Bi:V ratios | NiFeO_x_/BVO_6%_ | 4.8 | 4.2 | -- | ^[10]^ |
| H-treated and Mo-doped | Co-Ci/H,3% Mo: BiVO_4_ | 5.1 | 4.8 | -- | ^[11]^ |
| Oxygen vacancies | FeOOH/NiOOH/BVO | 4.5 | -- | -- | ^[12]^ |
| Micropillar Patterned | Micropillar Patterned BVO | 4.94 | 2.97 | 88.8 | ^[13]^ |
| Oxygen vacancies | Mo: BiVO_4_: 1-2 | 3.9 | 3.78 | 60 | ^[14]^ |
| Particle growth | RTP800-20s-50C/s-BiVO_4_/ NiFe(OH)_x_ | 3.2 | 2.7 | 82 | ^[15]^ |

Condition: the photocurrent density was tested at 1.23 V vs. RHE (mA/cm^2^)

**Table S4.** Fitting parameters for the TAS kinetics. The kinetics is fitted by a multiple-exponential function.

| Sample | τ_1_ (ps) | τ_2_ (ps) |
| --- | --- | --- |
| [010]-BVO | 100.55 | 100.55 |
| R-BVO | 46.46 | 56.79 |

**Reference**

[1] S. Wang, T. He, P. Chen, A. Du, K. K. Ostrikov, W. Huang, L. Wang, *Adv. Mater.* **2020**, *32*, e2001385.

[2] Z. Chen, T. F. Jaramillo, T. G. Deutsch, A. Kleiman-Shwarsctein, A. J. Forman, N. Gaillard, R. Garland, K. Takanabe, C. Heske, M. Sunkara, E. W. McFarland, K. Domen, E. L. Miller, J. A. Turner, H. N. Dinh, *J. Mater. Res.* **2010**, *25*, 3-16.

[3] B. Liu, X. Wang, Y. Zhang, L. Xu, T. Wang, X. Xiao, S. Wang, L. Wang, W. Huang, *Angew. Chem. Int. Ed.* **2023**, *62*, e202217346.

[4] D. Li, Y. Liu, W. Shi, C. Shao, S. Wang, C. Ding, T. Liu, F. Fan, J. Shi, C. Li, *ACS Energy Lett.* **2019**, *4*, 825-831.

[5] J. Narayan, B. C. Larson, *J. Appl. Phys.* **2003**, *93*, 278-285.

[6] M. Zhou, S. Zhang, Y. Sun, C. Wu, M. Wang, Y. Xie, *Chem.-Asian J.* **2010**, *5*, 2515-2523.

[7] J. H. Kim, J.-W. Jang, Y. H. Jo, F. F. Abdi, Y. H. Lee, R. van de Krol, J. S. Lee, *Nat. Commun.* **2016**, *7*, 13380.

[8] H. Lee, G.-S. Kang, H. Lim, H. Han, T. W. Kim, J.-H. Choi, D.-G. Choi, J.-Y. Jung, J.-H. Jeong, J. H. Park, J. Lee, *J. Mater. Chem. A* **2024**, *12*, 27246–27256.

[9] V. Nair, C. L. Perkins, Q. Lin, M. Law, *Energy Environ. Sci.* **2016**, *9*, 1412-1429.

[10] N. Yang, S. Zhang, Y. Xiao, Y. Qi, Y. Bao, P. Xu, S. Jin, F. Zhang, *Angew. Chem. Int. Ed.* **2023**, *62*, e202308729.

[11] J. H. Kim, Y. Jo, J. H. Kim, J. W. Jang, H. J. Kang, Y. H. Lee, D. S. Kim, Y. Jun, J. S. Lee, *Acs Nano* **2015**, *9*, 11820-11829.

[12] S. Feng, T. Wang, B. Liu, C. Hu, L. Li, Z. J. Zhao, J. Gong, *Angew. Chem. Int. Ed.* **2019**, *59*, 2044-2048.

[13] S. Ju, H. Kang, J. Jun, S. Son, J. Park, W. Kim, H. Lee, *Small* **2021**, *17*, e2006558.

[14] S. Wang, J. Jian, F. Li, Z. Zhang, X. Feng, Y. Shuang, Z. Ma, F. Wang, J. Wang, L. Zhang, L. Jia, H. Wang, *Chem. Eng. J.* **2024**, *496,* 154064.

[15] S. Wang, B. Liu, Q. Wang, Z. Gong, P. Zhang, T. Wang, J. Gong, *Adv. Funct. Mater.* **2024**, *34,* 2403019.
